# Relative role of life-history traits and historical factors in shaping genetic population structure of sardines (*Sardina pilchardus*)

**DOI:** 10.1186/1471-2148-7-197

**Published:** 2007-10-22

**Authors:** Elena G Gonzalez, Rafael Zardoya

**Affiliations:** 1 Departamento de Biodiversidad y Biología Evolutiva, Museo Nacional de Ciencias Naturales, CSIC, José Gutiérrez Abascal, 2; 28006 Madrid, Spain

## Abstract

**Background:**

Marine pelagic fishes exhibit rather complex patterns of genetic differentiation, which are the result of both historical processes and present day gene flow. Comparative multi-locus analyses based on both nuclear and mitochondrial genetic markers are probably the most efficient and informative approach to discerning the relative role of historical events and life-history traits in shaping genetic heterogeneity. The European sardine (*Sardina pilchardus*) is a small pelagic fish with a relatively high migratory capability that is expected to show low levels of genetic differentiation among populations. Previous genetic studies based on meristic and mitochondrial control region haplotype frequency data supported the existence of two sardine subspecies (*S. p. pilchardus *and *S. p. sardina*).

**Results:**

We investigated genetic structure of sardine among nine locations in the Atlantic Ocean and Mediterranean Sea using allelic size variation of eight specific microsatellite loci. Bayesian clustering and assignment tests, maximum likelihood estimates of migration rates, as well as classical genetic-variance-based methods (hierarchical AMOVA test and *R*_*ST *_pairwise comparisons) supported a single evolutionary unit for sardines. These analyses only detected weak but significant genetic differentiation, which followed an isolation-by-distance pattern according to Mantel test.

**Conclusion:**

We suggest that the discordant genetic structuring patterns inferred based on mitochondrial and microsatellite data might indicate that the two different classes of molecular markers may be reflecting different and complementary aspects of the evolutionary history of sardine. Mitochondrial data might be reflecting past isolation of sardine populations into two distinct groupings during Pleistocene whereas microsatellite data reveal the existence of present day gene flow among populations, and a pattern of isolation by distance.

## Background

Understanding the rather complex population structure and dynamics of marine pelagic fishes requires discerning the relative influence of life-history traits and historical processes in shaping present-day population patterns (e.g. [1-7]). Marine pelagic fishes exhibit great dispersal capability that enhances gene flow, as well as large effective population sizes that impose limitations to genetic drift (e.g. [8-11]). The combination of both life-history traits acts as major homogenizing force, which hampers genetic differentiation, and ultimately may lead to panmixia (e.g. [2,12,13]). In contrast, other life-history traits such as phylopatric behavior or local larval retention and recruitment act promoting isolation by distance, and local adaptations that eventually render low but significant levels of genetic differentiation in marine pelagic fish populations (e.g. [2,10,14]). Moreover, population structuring and dynamics of marine fishes are also heavily influenced by the physical peculiarities of the marine environment, where connectivity, and thus dispersal, is greatly dependant on ocean fronts and currents, as well as on bathymetry. For instance, the Agulhas current [15] seems to promote migration of bigeye tuna across the Cape of Good Hope from the Indian Ocean into the Atlantic Ocean (e.g. [9,16,17]) whereas the Almeria-Oran front [18] acts as a major barrier to gene flow between the Mediterranean Sea and the Atlantic Ocean for some species such as e.g. the mackerel [2], the anchovy [3] or the swordfish [7]. In addition, historical factors including past changes in the direction and sense of ocean currents, vicariant events caused by both climatic and eustatic sea level changes [7,9,19], as well as climate-associated periodical extinctions and recolonizations [20] have also decisively contributed to shaping present-day population genetic differentiation and geographic distribution.

Comparative analyses of both nuclear and mitochondrial genetic markers offer the best and most powerful approach to characterizing population genetic structure and diagnosing the evolutionary processes responsible for genetic differentiation in marine pelagic fishes (e.g. [6,17,21]). Therefore, genetic studies including both types of molecular markers are largely wanting.

The European sardine (*Sardina pilchardus*, Walbaum 1792) is a small pelagic fish that inhabits the coasts of the eastern North Atlantic Ocean (from the North Sea to Senegal), as well as the Mediterranean Sea, the Sea of Marmara, and the Black Sea [22,23]. Adults usually swim close to the littoral zone, and display daily vertical movement capacity [23,24]. Spawning occurs in open waters and larvae remain in plankton for long periods of time [24]. In spite of the relatively great dispersal capability of sardines both at the larval and adult stages, tagging and egg production data suggest that total inter annual displacement may be restricted by changes in the ocean water temperature and productivity, as well as by hydrogeographic boundaries [24-26]. Based on these life-history traits, sardine populations in close geographic proximity are expected to show modest genetic differentiation. That is the case in the Aegean Sea [27], the Spanish Mediterranean coast [28], and the Adriatic Sea [29]. At a larger scale, isolation by distance, and the existence of potential past or present-day barriers may promote higher levels of genetic differentiation.

Morphological studies based on gill raker counts and head length [22,30] found enough phenotypic variation to differentiate two subspecies, *S. p. pilchardus *(Eastern Atlantic Ocean, from the North Sea to Southern Portugal) and *S. p. sardina *(Mediterranean Sea and Northwest African coast). Although no private mitochondrial control region sequence haplotype could be found for each proposed subspecies, they were suggested to be genetically distinct based on significant pairwise haplotype frequency differences [31]. Moreover, some subspecies pairwise comparisons involving locations around the Atlantic Ocean region off the Gibraltar Strait showed no significant haplotype frequency differences, which suggested that this area could be a contact zone of both subspecies [31]. According to mitochondrial evidence, the well-known Almeria-Oran oceanographic front [18] between the Atlantic Ocean and the Mediterranean Sea is not a phylogenetic break for sardines.

The sardine is heavily fished all over its distribution with global catches of 1.600,000 tons per year (Fishery statistics 2003,[32]). In particular, Spain and Morocco are the countries with the largest captures (representing about the 77% of the total annual catch of sardines), and collapse of a sardine stock was reported off the Safi coast (Morocco) during the 1970s [33,34]. Population genetic and historical demographic analyses of sardines from Safi based on mitochondrial sequence data showed strong genetic differentiation of this population sample, and the signature of an early genetic bottleneck. The genetic singularity of the sardines at Safi (also detected with allozyme data [35]), could have enhanced the effects of the historical collapse of the sardine stock [31].

In this study, we analyzed allele size variation of eight polymorphic microsatellite loci in Atlantic and Mediterranean sardines. We used coalescent-based approaches for the estimation of the actual number of populations, and employed hierarchical AMOVA and isolation by distance tests to study population genetic differentiation. Our main objective was to explore whether microsatellites provide concordant genetic differentiation patterns with respect to mitochondrial control region sequence data [31]. Comparative analysis of mitochondrial and nuclear multilocus data were used to further understand the historical and contemporary (i.e. life-history) components of sardine population structure. In addition, we tested whether the genetic singularity of the Safi population sample could be confirmed with nuclear data, and whether any signature of a genetic bottleneck was detected in this or other population samples.

## Results

### Microsatellite diversity among loci

Microsatellite polymorphism levels were high at the eight genotyped loci with 41 to 94 alleles per locus (mean *N*_A _value ± standard deviation was 60 ± 18.60), and mean observed and expected heterozygosities of *H*_O _= 0.738 ± 0.13 and *H*_E _= 0.748 ± 0.14, respectively (Table 1). The inbreeding coefficient varied between 0.003 at locus *SAR2F *and 0.450 at locus *SAR1.12 *(mean *F*_IS _= 0.202 ± 0.18), and only two loci (*SAR2.18 *and *SARA2F*) were in Hardy-Weinberg (HW) equilibrium over all population samples (Table 1). Tests for linkage disequilibrium showed a very low (3.6%) number of significant pairwise comparisons, which suggests independence of all examined loci.

**Table 1:** Summary statistics for eight microsatellite loci and each population sample of Sardina pilchardus*

**Locus name**		***N*_A_**	***N*_S_**	***H*_E_**	***H*o**	***F*_IS_**
SAR1.5		41	22.03	0.944	0.834	**0.158**
SAR1.12		61	31.13	0.940	0.659	**0.450**
SAR2.18		41	24.72	0.949	0.775	0.009
SAR9		48	21.56	0.930	0.847	**0.090**
SAR19B3		60	28.84	0.956	0.562	**0.403**
SAR19B5		81	43.20	0.980	0.747	**0.156**
SARA2F		54	29.09	0.953	0.897	0.003
SARA3C		94	42.28	0.974	0.581	**0.349**
						
**Location**	***N***	***N*_A_**	***N*_S_**	***H*_E_**	***H*o**	***F*_IS_**

Dakhla	50	31.0	28.65	0.950	0.808	0.151
Tantan	47	30.7	28.58	0.950	0.775	0.187
Safi	50	29.4	26.55	0.948	0.742	0.219
Larache	50	29.4	27.20	0.951	0.682	**0.285**
Quarteira	47	27.4	25.99	0.944	0.728	**0.232**
Pasajes	49	30.0	28.15	0.953	0.726	**0.240**
Nador	47	29.2	27.08	0.946	0.726	**0.234**
Barcelona	45	27.5	26.45	0.944	0.692	0.269
Kavala	48	29.1	27.19	0.948	0.758	0.203

* *N *= population size; *N*_A _= total number of alleles per locus and mean number of alleles per population, respectively; *N*_S _= mean allelic richness standardized to the smallest sample size (42) using the rarefaction method of FSTAT 2.9.3 [66] per locus and population; mean expected (*H*_E_) and observed (*H*_O_) heterozygosities as well as mean *F*_IS _= Wright's statistics per locus and population. Bold *F*_IS _values are significant probability estimates after Bonferroni correction [69]

### Genetic diversity among sardine population samples and between subspecies

The amount of genetic variability was homogeneous among sardine population samples as indicated by the low standard deviations associated to the estimated mean number of alleles (*N*_A _= 29.3 ± 1.4), by mean allelic richness after rarefaction (*N*_S _= 27.3 ± 0.95), and by mean observed (*H*_O _= 0.747 ± 0.04) and expected (*H*_E _= 0.948 ± 0.00) heterozygosities (Table 1). The overall proportion of private alleles for the analyzed population samples was considerably high (32.1%). The inbreeding coefficient *F*_IS _within population samples across all loci was on average 0.224 ± 0.04. Sardine population samples at four locations (Larache, Quarteira, Pasajes, and Nador) showed significant mean *F*_IS _values, indicating significant departures from HW equilibrium due to homozygote excess (Table 1). A non-significant bimodal test indicated no evidence of unspecific locus amplification or genotyping errors, which could have resulted in null alleles. In addition, a null allele test based on expected homozygote and heterozygote allele size difference frequencies [36] detected that 55% of the pairwise comparisons presented HW disequilibrium mainly involving loci *SAR193B*, *SAR19B5 *and *SARA3C *(Additional file 1). We found that correcting for null allele frequencies [37] did not qualitatively affect the results (49% of the pairwise comparisons were still significant, data not shown). This suggests that putative null alleles had a very low effect on the average genetic diversity of our data, and hence the complete data set was included in further analyses.

The differences between *S. p. pilchardus *and *S. p. sardina *(as represented by Pasajes and the remaining population samples, respectively) genetic diversity measures were non-significant. The ANOVA test showed no differences for the mean number of alleles (*N*_A_) (*F*_1,7 _= 0.33, *P *= 0.58), the mean allelic richness after rarefaction (*N*_S_) (*F*_1,7 _= 0.85, *P *= 0.39), mean observed (*H*_O_) (*F*_1,7 _= 0.08, *P *= 0.78) and expected (*H*_E_) (*F*_1,7 _= 3.14, *P *= 0.12) heterozygosities, and the mean inbreeding coefficient (*F*_IS_) (*F*_1,7 _= 0.14, *P *= 0.71) between subspecies (Table 1).

### Estimation of the number of possible populations and assignment of individuals

**Figure 1:**
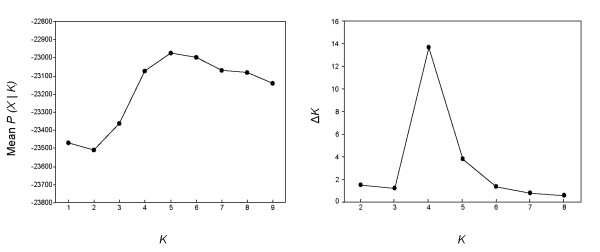
Number of sardine populations with the highest posterior probability expressed as the Δ*K*, for each of the nine assumed sardine populations (*K*). Δ*K *is calculated as the mean of the absolute values of the second derivative of L(*K*), (L" (*K*)) average over five runs divided by the standard deviation of L(*K*) [71].

Bayesian clustering analyses [38] detected the highest likelihood for the model with *K *= 5. However, the modal value of Δ*K *was shown at *K *= 4 (Fig. 1). A Bayesian inference under a Dirichlet process prior [39,40] estimated that the number of populations with the highest posterior probability was *K *= 3 (*P *= 1.0).

The probability of assignment of individuals to the four or five possible populations as inferred using Bayesian clustering analyses [38] was generally low (*P *< 0.8). The Bayesian assignment test correctly assigned 20.1% of the individuals to their own source location (22.4 % being the proportion of individuals that could not be assigned to any of the reference populations).

### Measures of genetic differentiation

The null hypothesis of no contribution of the Stepwise Mutation model (SMM; [41]) to genetic differentiation (ρ*R*_ST _= *F*_ST_) was rejected (*P *< 0.000) based on the multilocus data set (Table 2), suggesting that *R*_ST _should be preferred over *F*_ST _for the calculation of genetic differentiation between sardine population samples [42]. Three out of eight loci showed significant differences in the allele permutation test (Table 2). The significant global *R*_ST _test (0.024, *P *< 0.000, 95% C.I. 0.026 – 0.047) over all loci suggested population structuring in sardines. Of 36 pairwise comparisons, only nine comparisons involving Nador, Barcelona and Kavala locations revealed significant values after correction for multiple tests (Table 3). Interestingly, all pairwise comparisons between Pasajes (representing *S. s. pilchardus*) and the rest of the sampling sites (representing *S. s. sardina*) were non-significant.

**Table 2:** Summary statistics of the allele size permutation test [42] for each locus and the 95% confidence for the simulated *R*_ST _values*

Locus name	*F*_ST_	*ρR*_ST _(95% C.I.)	*R*_ST_
SAR1.5	0.0013	-0.0031 (-0.008 – 0.016)	-0.0013
SAR1.12	0.0022	0.0260 (0.208 – 0.385)	**0.0332**
SAR2.18	0.0079	0.0459 (0.089 – 0.281)	**0.0507**
SAR9	0.0011	-0.0033 (-0.008 – 0.014)	-0.0070
SAR19B3	0.0049	0.0013 (0.312 – 0.499)	0.0083
SAR19B5	0.0003	0.0054 (0.146 – 0.327)	0.0042
SARA2F	0.0048	0.0767 (-0.039 – 0.154)	**0.0785**
SARA3C	0.0031	0.0145 (0.310 – 0.496)	0.0204
Multilocus	0.0032	0.0182 (0.003 – 0.010)	**0.0242**

* Bold *R*_ST _values indicate a significant test (*R*_ST _> *ρR*_ST_) after Bonferroni correction [69]

**Table 3:** Multilocus estimates for *F*_*ST *_(below diagonal) and *R*_*ST *_(above diagonal) between sample pairs from eight microsatellite loci in common sardine

	Dakhla	Tantan	Safi	Larache	Quarteira	Pasajes	Nador	Barcelona	Kavala
Dakhla	--	0.008	0.016	0.006	0.012	0.022	**0.040**	**0.045**	**0.083**
Tantan	0.001	--	0.013	0.006	0.014	0.019	**0.037**	**0.045**	**0.073**
Safi	0.001	0.003	--	0.007	0.010	0.001	0.008	0.012	**0.043**
Larache	0.004	0.005	0.007	--	0.002	0.005	0.020	0.027	**0.048**
Quarteira	0.002	0.004	0.002	0.006	--	0.006	0.017	0.014	**0.035**
Pasajes	0.003	0.004	0.005	0.005	0.006	--	0.011	0.004	0.020
Nador	0.003	0.002	0.005	0.006	0.006	0.004	--	0.009	0.022
Barcelona	**0.008**	**0.008**	0.007	**0.010**	0.005	0.008	0.007	--	0.026
Kavala	**0.008**	**0.008**	**0.008**	0.007	**0.008**	0.006	0.007	0.008	--

Bold *P *values are significant after Bonferroni correction for 36 multiple tests (*P *< 0.0014) [69]

**Figure 2:**
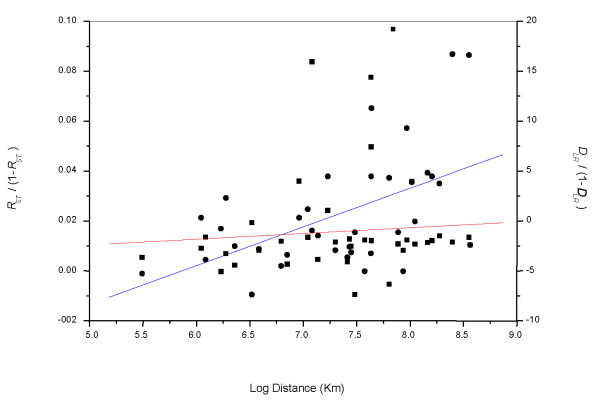
Genetic isolation by distance of all *S. pilchardus *population samples inferred from multilocus estimates of *R*_ST _(solid circles) and *D*_LR _(solid squares) genetic distances versus geographical distance (Mantel test). Correlation coefficients: for *R*_ST _r = 0.51, *R*^2 ^= 0.26, *P *< 0.009; for *D*_LR _r = 0.09, *R*^2 ^= 0.01, *P *= 0.59.

The hierarchical AMOVA revealed overall significant genetic structuring of the analyzed samples (*P *< 0.00) (Table 4). A two gene pool structure separating the subspecies *S. p. pilchardus *(Pasajes sampling site) versus *S. p. sardina *samples was not significant (*P *= 0.44). A possible *a priori *hypothesis of geographic structuring (organized as Atlantic Ocean versus Mediterranean Sea samples) was also not supported by the AMOVA (*P *= 0.07) (Table 4). The Atlantic Ocean versus Mediterranean Sea comparison was repeated excluding the Pasajes population sample, which could mask small genetic differentiation. Potential geographic structuring between the two areas remained not significant (Table 4). According to the Mantel test, correlation between genetic distance determined as *R*_ST _and geographical distance (log Km) was significant (correlation coefficient *r *= 0.51, *R*^2 ^= 0.26, *P *< 0.009) (Fig. 2). The Mantel test correlating *F*_ST _and geographic distances was not significant (not shown). Similarly, we found no significant correlation when using the Bayesian assignment *D*_LR _distances (correlation coefficient *r *= 0.09, *R*^2 ^= 0.01, *P *= 0.59) (Fig. 2).

**Table 4:** Analysis of molecular variance (AMOVA) of spatial genetic variation in common sardine for eight microsatellite loci *

Structure tested	Source of variation	F Statistics	Variance	Percentage of variation	*P*
(Dakhla, Tantan, Safi, Larache, Quarteira, Pasajes, Nador, Barcelona, Kavala)		*F*_*ST *_= 0.005			**0.00**
(Dakhla, Tantan, Safi, Larache, Quarteira, Nador, Barcelona, Kavala) vs. (Pasajes)	Among groups	*F*_*CT *_= 0.00	0.002	0.05	0.44
	Within groups	*F*_*SC *_= 0.005	0.020	0.54	**0.00**
	Within populations	*F*_*ST *_= 0.005	3.660	99.51	**0.00**
(Dakhla, Tantan, Safi, Larache, Quarteira, Pasajes) vs. (Nador, Barcelona, Kavala)	Among groups	*F*_*CT *_= 0.006	0.005	0.13	0.07
	Within groups	*F*_*SC *_= 0.005	0.017	0.47	**0.00**
	Within populations	*F*_*ST *_= 0.001	3.657	99.41	**0.00**
(Dakhla, Tantan, Safi, Larache, Quarteira) vs. (Nador, Barcelona, Kavala)	Among groups	*F*_*CT *_= 0.001	0.006	0.16	0.05
	Within groups	*F*_*SC *_= 0.005	0.017	0.46	**0.00**
	Within populations	*F*_*ST *_= 0.006	3.653	99.38	**0.00**

* Bold *P *numbers are significant values.

The Wilcoxon test detected recent bottlenecks in two population samples from the Mediterranean Sea corresponding to Nador and Kavala sampling sites (*P *two tails value of 0.031 for both tests), under the SMM model. No trace of genetic bottleneck was detected in Safi. Additionally, the test was performed using the Two Phase model (TPM; [43]) and the Infinite Allele model (IAM; [44]). In the first case, the test rendered non-significant results in all population samples. However Safi, Larache, Pasajes, Nador, and Kavala rendered significant results under the IAM.

### Levels and patterns of gene flow among populations

The estimates of the population size parameter (Θ) ranged from 0.38 to 0.81 (0.51 ± 0.13) (Table 5) and were translated to an average effective population size (*N*_*e*_) of 12,818 ± 325 sardine individuals (assuming a microsatellite mutation rate of 10^-4 ^per locus per generation [45]). Migration rates between population samples were all of the same order, and no preferential directionality of the migrants was observed. The mixed model-nested ANOVA test showed no significant variation of the number of emigrants and immigrants between the Atlantic Ocean and the Mediterranean Sea (*F*_1,8 _= 0.44, *P *= 0.53; *F*_1,8 _= 0.12, *P *= 0.74). Also the test rendered no significant variation of the number of emigrants among population samples (*F*_1,8 _= 0.00, *P *= 1.0). However a significant variation of immigrants among population samples (*F*_1,8 _= 3.14, *P *= 0.01) was detected. A one-way ANOVA was applied to test the null hypothesis of equal rate of immigrants between population samples. The analyses rendered a significant difference in the immigration rates among population samples (*F*_1,8 _= 3.67, *P *= 0.001), being Barcelona-Quarteira the only pairwise comparison that was significantly different (t > 1.998).

**Table 5:** Maximum likelihood estimates of the population size, Θ (Θ = 4 × effective population size, *N*_*e *_× mutation rate, μ per generation and site) and the scaled migration rate, *M *(*M *= immigration rate per generation *m*/μ) for all population samples of *Sardina pilchardus*. Θ values are displayed on the diagonal. All values are within the bounds of 95% interval of confidence

	Migration rate (*M*)^a^									
		
Location	Dakhla	Tantan	Safi	Larache	Quarteira	Pasajes	Nador	Barcelona	Kavala	N_e_^b^
	
Dakhla	0.62	8.02	9.60	7.76	6.51	11.88	13.92	9.88	7.62	15500
Tantan	8.77	0.81	13.74	9.46	8.54	10.79	11.38	5.85	8.85	20346
Safi	11.40	11.23	0.48	13.85	14.67	13.22	10.52	5.76	9.10	12012
Larache	8.70	10.46	15.60	0.50	13.33	10.75	10.87	6.03	7.73	12550
Quarteira	4.35	7.81	9.81	8.04	0.42	18.24	17.72	14.35	13.84	10568
Pasajes	11.88	12.51	12.58	9.35	16.52	0.42	14.92	8.60	11.66	10472
Nador	11.60	8.54	9.60	8.16	16.22	16.78	0.49	7.41	12.22	12280
Barcelona	10.34	3.90	9.00	10.70	20.47	10.58	10.61	0.38	8.60	9428
Kavala	9.52	6.57	7.79	5.21	11.86	10.60	12.88	6.35	0.49	12208

^a ^Rows and columns are donor and recipient populations, respectively. ^b ^*N*_*e *_was calculated assuming μ = 10^-4 ^per locus and per generation

## Discussion

The study of population genetic variation of marine pelagic fish species has proven to be particularly challenging because of the biological peculiarities of these fishes including large effective population sizes and high dispersal capacities, as well as because of the apparent lack of physical barriers to gene flow in the marine realm [6,46-48]. Mitochondrial DNA is maternally inherited, lacks recombination, and shows relatively fast evolutionary rates, which make this molecular marker particularly suitable for inferring phylogeographic patterns [49]. This molecular marker is particularly appropriate for detecting historical vicariant or genetic bottleneck events, and has been very useful in describing present day phylogeography of taxa with relatively low dispersal capacity [49]. However, mitochondrial genetic variation is less helpful when tackling questions on present-day genetic structuring of taxa with large population sizes and high levels of gene flow within their distribution such as marine pelagic fishes (e.g. [6,21]). Microsatellites are nuclear markers with higher mutation rates [50] that have proved to be more efficient and informative for detecting fine-scale population structure in marine pelagic fishes [17,21,51]. Overall, comparative analyses of nuclear and mitochondrial data should provide insights not achieved by each type of data separately, and should help in disentangling historical versus ecological factors involved in shaping contemporary population genetic structure of marine pelagic fishes [21].

### Population genetic structure in sardines

The eight species-specific microsatellite loci used in this study showed high levels of polymorphism [52] and no significant linkage disequilibrium. All but two of the analyzed loci showed departures from HW equilibrium expectations due to homozygote excess. The null allele test [36] indicated that these departures could be due to the presence of null alleles, which seem to be rather common in large marine fish populations [53]. Nevertheless, since adjusting frequencies to take into account null alleles did not affect inbreeding coefficient estimates, all loci were used in the analyses.

Overall *R*_*ST *_detected weak but significant genetic structuring among sardine population samples. Pairwise estimates of *R*_*ST *_varied between 0.001 and 0.083, and were of the same level of magnitude to those reported for other marine fishes [53-56]. These relatively low *R*_*ST *_values could be attributed to high levels of size homoplasy, as expected when using polymorphic microsatellites with high mutation rates in species with large effective population sizes [53,57,58]. However, the observed relatively high number (32.1%) of private alleles, and their even distribution among population samples indicate that allele sharing between sardines at the different locations is rather limited and thus, that the effects of size homoplasy are minimal. Alternatively, it is more likely that the high levels of locus polymorphism are the ones responsible of only detecting weak genetic structuring [53,58].

The difficulty in detecting genetic structuring is further evidenced by Bayesian clustering and assignment tests, as well as by hierarchical AMOVA and migration rate analyses. Although the different assayed Bayesian clustering analyses agree in rejecting the null hypothesis of panmixia, they failed to predict the exact number of inferred populations, which ranges from 3 to 5. Furthermore, assignment of individuals to the inferred populations was poor regardless of the method used. In addition, none of the tested *a priori *hypotheses of genetic structuring rendered significant results in the AMOVA. Maximum likelihood estimates of migration rates showed that gene flow among population samples is high and even. All these results together support that sardine population samples are acting as a single significant evolutionary unit. The Mantel test detected positive and significant correlation between genetic differentiation (only when using *R*_ST_) and geographical distance suggesting that a model of isolation by distance could explain the subtle genetic structuring of sardines within the evolutionary unit. Isolation by distance seems to be a rather common pattern in small-medium pelagic marine fishes (e.g. [2,19,51]). It is important to note here that temporal replicates at the studied locations are needed to test whether the observed population genetic patterns are stable over time.

### Relative effects of life-history traits and historical factors on genetic differentiation in sardines

All significant *R*_*ST *_pairwise comparisons involved Mediterranean Sea versus Central Atlantic Ocean population samples. Theses results could reflect the existence of a phylogeographic discontinuity between the Atlantic Ocean and Mediterranean Sea, around the Gibraltar Strait and the Almeria-Oran front, as it has been postulated previously for different marine pelagic fish species (e.g. [2,3,7]). However, this hypothesis was rejected for sardines at the nuclear level because the hierarchical AMOVA failed to detect significant geographical structuring between the Atlantic and the Mediterranean sardine population samples, and high and even migration rates were observed between both basins. These results are congruent with those derived from population genetic analyses based on mitochondrial control region sequence data that also failed to find a barrier to gene flow for sardines at the Atlantic Ocean and the Mediterranean Sea [31]. The inferred genetic pattern for sardine is in agreement with the present-day gene flow exhibited by other marine pelagic fish species such as e.g. *Scomber japonicus *[2] or *Thunnus thynnus *[7] through the Atlantic-Mediterranean transition. The fact that the Gibraltar Strait and the Almeria-Oran front may or not act as barrier to gene flow for different marine pelagic species has been attributed to differences in life-history traits (e.g. dispersal capacity [2]) and for other marine fish species due to the existence of distinct past demographical events (e.g. bottlenecks [7]). More comparative studies on the biology and population dynamics of marine pelagic fishes distributed at both sides of the Gibraltar Strait, as well as additional population genetic analyses including temporal series are needed to further understand the factors that promote or prevent gene flow of these species across the Atlantic-Mediterranean transition.

The existence of two different subspecies (*S. p. pilchardus *and *S. p. sardina*) as previously reported based on meristic studies [22,30], and mitochondrial control region sequence haplotypes frequency differences [31] was not supported by population genetic analyses (*R*_*ST *_pairwise comparisons, AMOVA test, and estimations of migration rates) based on microsatellite data. However, these results need to be taken with caution since one of the subspecies (*S. p. pilchardus*) was only represented by a single location (Pasajes). A more thorough sampling of sardine at North Atlantic locations would be mandatory to further test the validity of the two subspecies using microsatellite allele frequency data.

The discordant genetic structuring patterns inferred based on mitochondrial and microsatellite data could indicate that the two different classes of molecular markers may be reflecting different and complementary aspects of the evolutionary history of sardine. The significant genetic structuring evidenced by mitochondrial data might be reflecting past isolation of sardine populations into two distinct groupings during Pleistocene [31]. Afterwards, sardine populations expanded and secondary contact was re-established around the Gibraltar Strait. Microsatellite data reveal the existence of a present day single evolutionary unit that shows weak genetic structuring due to isolation by distance. At micro geographical scale, genetic drift is supposed to overcome gene flow as geographical distance increases [59] because of the effect of different life-history traits such as e.g. larval retention, homing behavior, or reduced dispersal capacity, that need to be further studied in sardines.

Periodic population extinctions and recolonizations at the regional level are common in sardines and other clupeids and may be responsible for the shallow coalescence of mitochondrial genealogies [20]. In this regard, mitochondrial and nuclear markers exhibit different performance in detecting instances of genetic bottlenecks. Mitochondrial control region sequence data support the existence of a past (Pleistocene) genetic bottleneck of sardines in Safi that is only detected at the nuclear level using the IAM. In addition, analyses of microsatellite data under both the SMM and IAM revealed potential genetic bottlenecks at Kavala and Nador, which would be too recent to be detected by mitochondrial data.

Different types of genetic markers occasionally may render contrasting population genetic structure patterns for a given species [21,60]. In some instances, discordance among marker classes may result from methodological biases, which when appropriately corrected allow obtaining reconciled patterns [21,60]. In other cases, conflicting results in describing population genetic structure may arise from the differential effects of genetic drift and mutation on a marker class [21]. In such cases, discordance could be interpreted as a source of alternative and complementary information useful for investigating how evolutionary processes at different time scales shape patterns of genetic heterogeneity. In this study, the comparison of two classes of molecular markers with different mutation rates and modes of inheritance has allowed us to gain complementary and broader insights on sardine historical and contemporary population genetics and dynamics, which ultimately could serve to improve fishery management of this commercially important marine pelagic fish species.

## Conclusion

The discordant genetic structuring patterns inferred based on mitochondrial and microsatellite data appear to be pointing to complementary aspects of the evolutionary history of sardine. Past isolation of sardine populations into two distinct groupings is supported by mitochondrial data whereas current gene flow within a single evolutionary unit and a weak genetic structuring due to isolation by distance are evidenced by microsatellite data. This study shows that only the combination of molecular markers with different modes of inheritance and mutation rates is able to disentangle the complex patterns of population structure and dynamics of a small marine pelagic fish such as the sardine.

## Methods

### Sample collection

**Figure 3:**
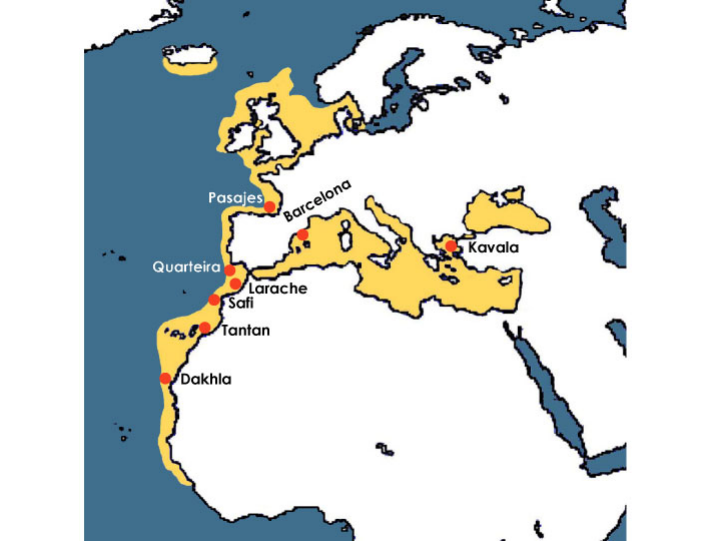
Locations of sardine samples collected in the Atlantic Ocean and Mediterranean Sea (red circles). The yellow colored area shows the distribution of *S. pilchardus*. Details for sample sizes are listed in Table 1.

We extended the sample collection of a previous study [61] from about 25–30 to nearly 50 mature sardine specimens per landing port. Overall, population genetic analyses included 433 individuals from six localities (Dakhla, Tantan, Safi, Larache, Quarteira and Pasajes) in the Atlantic Ocean (*N *= 293) and three localities (Nador, Barcelona and Kavala) in the Mediterranean Sea (*N *= 140) (Fig. 3). The sardines from the Pasajes location were assigned to the subspecies *S. p. pilchardus *based on distribution area, and mitochondrial haplotypes frequencies. The sardines from the remaining locations were assigned to the subspecies *S. p. sardina *based on the same criteria.

### Microsatellite genotyping

Genomic DNA of newly analyzed specimens was extracted from fresh muscle following standard phenol-chloroform procedures as previously reported [61]. Specific polymorphic microsatellites (*SAR1.5*, *SAR1.12*, *SAR2.18*, *SAR9*, *SAR19B3*, *SAR19B5*, *SARA2F *and *SARA3C*) of *S. pilchardus *were PCR amplified following optimized reaction conditions [62]. Forward primers were labeled with fluorescent dye (Invitrogen), and PCR amplified products were genotyped on an ABI 3730 automated sequencer (Applied Biosystems). Data collection and sizing of alleles were carried out using GeneMapper v3.7 software (Applied Biosystems). Approximately 10% of the samples were re-run to assess repeatability in scoring.

### Statistical analyses

Microsatellite genetic diversity was quantified per locus and per sampling site as the observed and expected heterozygosities [63], number of alleles (*N*_A_), and number of alleles standardized to those of the population sample with the smallest size (*N*_S_) [64], using both GENETIX 4.02 [65] and FSTAT 2.9.3 [66]  (Additional file 2). Deviations from HW equilibrium (by estimating the inbreeding coefficient, *F*_IS_) and linkage disequilibrium for each locus and sardine sampling site were assessed using GENEPOP version3.3 [67]. Significance of both analyses was tested with a Markov chain Monte Carlo (MCMC) that was run for 1000 batches of 2000 iterations each, with the first 500 iterations discarded before sampling [68]. *P *values from multiple comparisons were corrected using a Bonferroni correction [69]. Significant differences of genetic diversity measures between *S. pilchardus *subspecies were tested using a one-way ANOVA test.

A bimodal test for each locus and sampling site was performed to detect possible genotyping errors due to preferential amplification of one of the two alleles, misreading of bands or transcription errors, using the program DROPOUT [70]. Additionally, MICRO-CHECKER v2.23 [36], was used to explore the existence of null alleles, and to evaluate their impact on the estimation of genetic differentiation.

### Genetic and spatial variation between populations

Several alternative methods were used to determine sardine population genetic structure. The program STRUCTURE 2.0 [38] uses a model-based Bayesian clustering approach to determine the number of populations (*K*) with the highest posterior probability and to estimate admixture proportions. Simulations were conducted using an admixture model and correlated allele frequencies between populations (MCMC consisted of 5 × 10^5 ^burn-in iterations followed by 2 × 10^6 ^sampled iterations). Additionally, the inference of the best value of *K *was also based on the modal value of Δ*K *[71]. The range of possible tested *K*s was from one to nine, and five trial runs of STRUCTURE were carried out for each putative *K*.

The program STRUCTURAMA [72] infers population genetic structure from genetic data by allowing the number of populations to be a random variable that follows a Dirichlet process prior [39,40]. We run 1 × 10^6 ^MCMC cycles, and we let α (the prior mean of the number of populations) be a random variable. The first 1 × 10^5 ^cycles were discarded as burn-in.

We finally applied a Bayesian assignment test as implemented in the program GENECLASS 2.0 [73], which provides the probability for each individual of belonging to the reference population. The computation followed the partial exclusion method [74], and simulation consisted of 10,000 individuals.

The relative contributions of mutation and genetic drift to genetic differentiation of sardine populations could be determined by comparing the variance in allelic identity (*F*_ST_, IAM [44]) and allelic size (*R*_ST_, SMM [41]). The program SPAGEDI 1.1 [75] generates a simulated distribution of *R*_*ST *_values (ρ*R*_*ST*_) for testing the null hypothesis of no contribution of SSM to genetic differentiation (ρ*R*_ST _= *F*_ST_), and the alternative hypothesis that genetic differentiation is caused mainly by SMM-like mutation (ρ*R*_ST _> *F*_ST_,) [42]. The test rendered a significant result (*P *< 0.000), and thus, further analyses of genetic differentiation between samples were mostly based on *R*_ST _pairwise comparisons, as estimated by the program RST-CALC [76].

To determine the amount of genetic variability partitioned within and among populations, an analysis of molecular variance (AMOVA) [77] was performed with ARLEQUIN v3.0 [78]. For all calculations, significance was assessed by 20,000 permutations, and reported *P*-values were Bonferroni adjusted [69]. The Mantel test was used to test correlation between geographical and genetic distances as implemented in GENEPOP version3.3 [67]. The logarithm of geographical distance in kilometers was regressed against either *R*_*ST *_as estimated in RST-CALC [76] or genetic distances based on Bayesian assignment values (*D*_LR_) as computed in SPASSIGN [79].

To detect possible genetic bottlenecks (i.e. significant heterozygote excess) in any of the analyzed population samples, we assumed the SMM, IAM, and TPM, and applied the Wilcoxon sign-rank test as implemented in the software BOTTLENECK [80].

### Gene flow among sardine populations

The program MIGRATE v 2.1.0 [81] was used to infer the population size parameter Θ (i.e. 4 *N*_*e*_*μ*, were *N*_*e *_is the effective population size and *μ *is the mutation rate per site) and the migration rate, *M *(*M *= m/*μ*, were m is the immigration rate per generation) among sardine population samples based on the maximum likelihood method [82]. A subset of 20 individuals per population sample was analyzed due to computational constraints. The analyses were carried under the SMM. *F*_*ST *_estimates and a UPGMA tree were used as starting parameters for the estimation of Θ and *M*. The MCMC run consisted of ten short and two long chains with 5,000 and 50,000 recorded genealogies respectively, after discarding the first 100,000 genealogies (burn-in). One of every 20 and 200 reconstructed genealogies was sampled for the short and long chains, respectively. To test the null hypothesis that the number of emigrants/ immigrants between the Atlantic Ocean and Mediterranean Sea has equal rates, a nested mixed-model ANOVA was performed using two variables (basin and location of origin), with emigrant and immigrant rates as repeated measurements.

## Competing interests

The author(s) declares that there are no competing interests.

## Authors' contributions

EGG carried out the DNA genotyping, performed the statistical analyses and drafted the manuscript. RZ conceived the study, supervised the genetic studies, and contributed to the writing of the manuscript. Both authors read and approved the final manuscript.

## Supplementary Material

Additional file 1 Summary statistics for eight microsatellite loci of *Sardina pilchardus *population samples. The data provided summarizes the statistical analyses (*N *= sample size, *N*_A _= number of alleles per locus, *N*_S _= number of alleles per locus standardized to the smallest sample size (42), expected (*H*_E_) and observed (*H*_O_) heterozygosities and *F*_IS _= Wright's statistics) for each locus and sampling site. Click here for file

Additional file 2 Allele frequency and allele size for eight microsatellite loci and each sample of *Sardina pilchardus*. The table shows the allele frequency and allele size values for each locus and sampling site. Click here for file
