# Supplementary material for: Relative role of life-history traits and historical factors in shaping genetic population structure of sardines (Sardina pilchardus)
Source: BMC Evol Biol. 2007 Oct 22;7:197. doi: 10.1186/1471-2148-7-197 (PMC2204010; doi:10.1186/1471-2148-7-197)
Supplement: Additional file 1 — Summary statistics for eight microsatellite loci of Sardina pilchardus population samples. The data provided summarizes the statistical analyses (N = sample size, NA = number of alleles per locus, NS = number of alleles per locus standardized to the smallest sample size (42), expected (HE) and observed (HO) heterozygosities and FIS = Wright's statistics) for each locus and sampling site. [file 1471-2148-7-197-S1.pdf]

Additional Table 1: Summary statistics for eight microsatellite loci of *Sardina pilchardus* samples *

		Locus							
Location		SAR1.5	SAR1.12	SAR2.18	SAR9	SAR19B3	SAR195B5	SARA2F	SARA3C
Dakhla	N	50	50	50	50	45	47	46	46
	N_A	25	33	22	23	27	45	33	40
	N_S	23.93	30.30	20.67	21.93	26.51	42.55	31.74	38.60
	H_E	0.948	0.933	0.933	0.938	0.956	0.976	0.944	0.973
	Ho	0.880	0.660	0.940	0.920	0.800	0.830	0.891	0.544
	F_{IS}	0.072	**0.295**	-0.008	0.020	**0.165**	**0.151**	0.056	**0.444**
Tantan	N	47	47	47	46	46	46	46	43
	N_A	19	32	22	23	30	42	34	44
	N_S	18.67	30.53	21.13	22.20	28.84	40.28	32.90	43.55
	H_E	0.941	0.931	0.922	0.932	0.956	0.976	0.964	0.981
	Ho	0.766	0.787	0.872	0.826	0.826	0.674	0.957	0.488
	F_{IS}	**0.188**	0.156	0.055	0.115	0.137	**0.312**	0.008	**0.505**
Safi	N	50	50	50	50	50	49	50	50
	N_A	21	25	25	19	28	45	29	43
	N_S	20.14	24.04	23.95	17.83	26.59	41.81	27.11	39.63
	H_E	0.941	0.942	0.951	0.902	0.955	0.979	0.948	0.969
	Ho	0.860	0.560	0.740	0.880	0.520	0.816	0.960	0.600
	F_{IS}	0.087	**0.408**	**0.223**	0.025	**0.458**	**0.167**	-0.013	**0.383**
Larache	N	50	50	50	50	48	48	47	48
	N_A	22	38	22	26	23	39	26	39
	N_S	21.23	35.05	21.23	24.16	22.53	37.31	25.11	37.16
	H_E	0.938	0.962	0.938	0.940	0.937	0.972	0.952	0.971
	Ho	0.800	0.760	0.540	0.860	0.500	0.667	0.787	0.517
	F_{IS}	**0.149**	**0.212**	**0.427**	0.086	**0.469**	**0.317**	**0.175**	**0.445**
Quarteira	N	47	46	47	47	44	47	46	46
	N_A	23	31	26	20	22	36	25	36
	N_S	22.41	29.81	25.22	19.40	21.72	34.35	24.45	34.97
	H_E	0.947	0.922	0.955	0.913	0.937	0.972	0.952	0.971
	Ho	0.809	0.848	0.915	0.745	0.930	0.970	0.949	0.968
	F_{IS}	0.148	0.081	0.043	0.186	**0.563**	**0.367**	0.108	**0.351**
Pasajes	N	49	49	49	49	48	47	48	48
	N_A	22	30	28	25	26	40	31	38
	N_S	21.40	28.39	26.57	23.74	25.09	38.59	29.32	36.22
	H_E	0.946	0.943	0.945	0.934	0.956	0.979	0.954	0.969
	Ho	0.857	0.694	0.592	0.755	0.542	0.766	0.875	0.729
	F_{IS}	0.095	**0.266**	**0.376**	**0.193**	**0.436**	**0.219**	0.083	**0.249**
Nador	N	47	47	47	47	47	46	45	46
	N_A	19	26	24	20	30	46	29	40
	N_S	18.75	24.96	23.23	19.23	28.76	43.93	28.24	38.77
	H_E	0.930	0.923	0.929	0.923	0.954	0.980	0.951	0.976
	Ho	0.872	0.596	0.766	0.872	0.511	0.783	0.911	0.500
	F_{IS}	0.063	**0.357**	0.177	0.055	**0.467**	**0.204**	0.042	**0.491**
Barcelona	N	45	44	43	45	44	42	43	45
	N_A	27	27	22	22	24	42	23	33
	N_S	26.31	26.53	21.93	21.53	23.68	42.00	22.84	32.03
	H_E	0.946	0.914	0.948	0.936	0.948	0.981	0.931	0.950
	Ho	0.867	0.500	0.674	0.911	0.386	0.738	0.907	0.556
	F_{IS}	0.085	**0.456**	**0.291**	0.027	**0.595**	**0.250**	0.026	**0.418**
Kavala	N	48	48	48	48	46	47	47	48
	N_A	22	27	25	23	33	45	24	34
	N_S	21.11	25.91	24.16	22.20	31.57	42.95	23.24	32.36
	H_E	0.939	0.943	0.946	0.938	0.942	0.982	0.939	0.956
	Ho	0.792	0.521	0.938	0.854	0.565	0.982	0.936	0.625
	F_{IS}	**0.158**	**0.450**	0.009	0.090	**0.403**	**0.156**	0.003	**0.349**

* N = sample size, N_A = number of alleles per locus, N_S = number of alleles per locus standarized to the smallest sample size (42), expected (H_E) and observed (Ho) heterozygosities , F_{IS} = Wright's statistics. Bold F_{IS} values are significant probability estimates after Bonferroni correction (Rice 1989)
