# Supplementary material for: Relative role of life-history traits and historical factors in shaping genetic population structure of sardines (Sardina pilchardus)
Source: BMC Evol Biol. 2007 Oct 22;7:197. doi: 10.1186/1471-2148-7-197 (PMC2204010; doi:10.1186/1471-2148-7-197)
Supplement: Additional file 2 — Allele frequency and allele size for eight microsatellite loci and each sample of Sardina pilchardus. The table shows the allele frequency and allele size values for each locus and sampling site. [file 1471-2148-7-197-S2.pdf]

Additional Table 2: Allele frequency and allele size for eight microsatellite loci and each sample of *Sardina pilchardus* *

			Population									
Locus	N_A	Allele size	Dakhla	Tantan	Safi	Larache	Quarteira	Pasajes	Nador	Barcelona	Kavala	Overall
SAR1.5	1	119	0.00	0.00	0.00	0.00	0.00	0.00	0.00	0.00	0.01	0.00
	2	121	0.00	0.00	0.00	0.03	0.00	0.00	0.00	0.00	0.01	0.00
	3	123	0.00	0.00	0.00	0.00	0.00	0.00	0.00	0.01	0.00	0.00
	4	125	0.00	0.00	0.00	0.00	0.00	0.02	0.00	0.02	0.00	0.00
	5	127	0.00	0.01	0.04	0.00	0.01	0.00	0.00	0.02	0.00	0.01
	6	128	0.03	0.00	0.00	0.02	0.01	0.04	0.00	0.00	0.00	0.01
	7	129	0.05	0.04	0.01	0.05	0.02	0.04	0.05	0.03	0.05	0.04
	8	131	0.03	0.05	0.08	0.04	0.09	0.04	0.04	0.11	0.07	0.06
	9	132	0.08	0.06	0.10	0.13	0.06	0.09	0.04	0.01	0.11	0.08
	10	134	0.05	0.03	0.08	0.01	0.07	0.09	0.11	0.03	0.03	0.06
	11	136	0.10	0.09	0.06	0.08	0.06	0.09	0.03	0.11	0.04	0.07
	12	138	0.03	0.06	0.01	0.07	0.04	0.10	0.04	0.09	0.06	0.06
	13	140	0.11	0.12	0.10	0.04	0.11	0.05	0.09	0.10	0.08	0.09
	14	142	0.07	0.10	0.08	0.11	0.02	0.08	0.14	0.06	0.13	0.09
	15	144	0.06	0.09	0.08	0.05	0.06	0.04	0.13	0.07	0.08	0.07
	16	146	0.09	0.04	0.02	0.07	0.06	0.03	0.10	0.03	0.07	0.06
	17	147	0.00	0.00	0.00	0.00	0.00	0.00	0.00	0.01	0.00	0.00
	18	148	0.03	0.06	0.04	0.10	0.06	0.07	0.02	0.02	0.05	0.05
	19	150	0.03	0.07	0.03	0.06	0.06	0.03	0.01	0.01	0.03	0.04
	20	152	0.04	0.03	0.06	0.02	0.09	0.04	0.03	0.04	0.02	0.04
	21	154	0.06	0.02	0.08	0.02	0.02	0.03	0.04	0.02	0.05	0.04
	22	156	0.02	0.04	0.05	0.03	0.02	0.01	0.02	0.06	0.03	0.03
	23	158	0.02	0.00	0.00	0.00	0.04	0.03	0.02	0.02	0.01	0.02
	24	160	0.00	0.00	0.00	0.00	0.00	0.01	0.00	0.00	0.00	0.00
	25	161	0.02	0.00	0.00	0.02	0.01	0.01	0.00	0.00	0.00	0.01
	26	162	0.02	0.05	0.03	0.01	0.02	0.03	0.04	0.04	0.01	0.03
	27	164	0.01	0.01	0.01	0.02	0.02	0.00	0.03	0.01	0.01	0.01
	28	166	0.00	0.00	0.00	0.00	0.00	0.00	0.00	0.01	0.00	0.00
	29	167	0.00	0.00	0.00	0.00	0.01	0.00	0.00	0.01	0.00	0.00
	30	169	0.01	0.00	0.00	0.01	0.00	0.00	0.00	0.00	0.00	0.00
	31	170	0.00	0.00	0.00	0.00	0.00	0.00	0.01	0.01	0.00	0.00
	32	171	0.00	0.01	0.00	0.00	0.01	0.00	0.00	0.00	0.00	0.00
	33	172	0.01	0.00	0.02	0.00	0.00	0.01	0.00	0.00	0.00	0.00
	34	174	0.00	0.00	0.00	0.00	0.00	0.00	0.00	0.00	0.01	0.00
	35	175	0.01	0.00	0.00	0.00	0.00	0.00	0.00	0.00	0.00	0.00
	36	176	0.00	0.00	0.00	0.00	0.00	0.00	0.00	0.01	0.01	0.00
	37	177	0.00	0.00	0.01	0.00	0.00	0.00	0.00	0.00	0.00	0.00
	38	180	0.00	0.00	0.01	0.00	0.00	0.00	0.00	0.00	0.00	0.00
	39	181	0.01	0.00	0.00	0.01	0.00	0.00	0.00	0.00	0.00	0.00
	40	208	0.01	0.00	0.00	0.00	0.00	0.00	0.00	0.00	0.00	0.00
	41	224	0.00	0.00	0.00	0.00	0.00	0.00	0.00	0.01	0.00	0.00
SAR1.12	1	142	0.00	0.00	0.00	0.00	0.00	0.00	0.01	0.00	0.00	0.00
	2	156	0.00	0.00	0.02	0.00	0.00	0.00	0.04	0.00	0.00	0.01
	3	166	0.01	0.01	0.00	0.00	0.00	0.00	0.02	0.05	0.04	0.01
	4	168	0.00	0.00	0.00	0.04	0.02	0.00	0.00	0.00	0.00	0.01
	5	170	0.00	0.00	0.00	0.02	0.00	0.02	0.01	0.02	0.00	0.01
	6	174	0.00	0.00	0.00	0.00	0.00	0.02	0.01	0.00	0.00	0.00
	7	180	0.00	0.00	0.00	0.02	0.00	0.00	0.00	0.00	0.00	0.00
	8	182	0.00	0.02	0.00	0.00	0.00	0.00	0.00	0.00	0.03	0.01
	9	184	0.00	0.00	0.02	0.02	0.00	0.00	0.02	0.00	0.00	0.01
	10	186	0.01	0.00	0.00	0.02	0.03	0.02	0.00	0.00	0.01	0.01

	11	188	0.03	0.00	0.01	0.00	0.01	0.00	0.02	0.01	0.04	0.01
	12	190	0.01	0.02	0.02	0.03	0.00	0.03	0.01	0.01	0.02	0.02
	13	192	0.06	0.03	0.07	0.05	0.02	0.07	0.07	0.09	0.07	0.06
	14	193	0.00	0.01	0.00	0.00	0.00	0.00	0.00	0.00	0.00	0.00
	15	194	0.03	0.03	0.05	0.01	0.01	0.02	0.04	0.00	0.00	0.02
	16	196	0.02	0.03	0.05	0.05	0.01	0.02	0.02	0.10	0.05	0.04
	17	198	0.19	0.20	0.13	0.06	0.22	0.13	0.19	0.24	0.14	0.16
	18	200	0.09	0.13	0.11	0.12	0.13	0.14	0.10	0.09	0.13	0.11
	19	202	0.00	0.01	0.07	0.04	0.01	0.03	0.05	0.02	0.03	0.03
	20	203	0.01	0.00	0.00	0.00	0.00	0.00	0.00	0.00	0.00	0.00
	21	204	0.12	0.09	0.11	0.05	0.10	0.07	0.14	0.01	0.01	0.08
	22	206	0.03	0.01	0.04	0.01	0.03	0.04	0.02	0.03	0.04	0.03
	23	207	0.00	0.00	0.00	0.00	0.00	0.00	0.00	0.01	0.00	0.00
	24	208	0.01	0.03	0.05	0.01	0.00	0.01	0.00	0.02	0.00	0.02
	25	209	0.03	0.00	0.00	0.00	0.00	0.00	0.00	0.00	0.00	0.00
	26	210	0.02	0.01	0.03	0.08	0.01	0.05	0.06	0.02	0.09	0.04
	27	212	0.04	0.02	0.00	0.00	0.02	0.07	0.00	0.00	0.00	0.02
	28	214	0.01	0.00	0.02	0.01	0.03	0.02	0.00	0.03	0.02	0.02
	29	216	0.05	0.01	0.02	0.03	0.02	0.01	0.03	0.05	0.01	0.03
	30	218	0.01	0.00	0.00	0.02	0.00	0.04	0.02	0.01	0.02	0.01
	31	220	0.00	0.02	0.02	0.00	0.03	0.02	0.01	0.02	0.00	0.01
	32	222	0.01	0.03	0.03	0.02	0.00	0.01	0.00	0.01	0.05	0.02
	33	224	0.02	0.04	0.03	0.00	0.03	0.01	0.02	0.01	0.01	0.02
	34	226	0.01	0.02	0.00	0.01	0.00	0.00	0.01	0.02	0.00	0.01
	35	228	0.01	0.02	0.03	0.04	0.01	0.02	0.02	0.02	0.02	0.02
	36	230	0.01	0.02	0.00	0.01	0.05	0.00	0.00	0.00	0.00	0.01
	37	232	0.03	0.04	0.03	0.01	0.02	0.00	0.00	0.00	0.02	0.02
	38	234	0.04	0.03	0.00	0.01	0.02	0.03	0.00	0.02	0.03	0.02
	39	236	0.00	0.02	0.00	0.01	0.02	0.00	0.01	0.01	0.00	0.01
	40	237	0.00	0.00	0.00	0.00	0.00	0.01	0.00	0.00	0.00	0.00
	41	238	0.01	0.01	0.00	0.01	0.01	0.01	0.00	0.00	0.01	0.01
	42	240	0.00	0.01	0.00	0.00	0.00	0.02	0.00	0.01	0.02	0.01
	43	242	0.00	0.01	0.01	0.02	0.02	0.00	0.00	0.00	0.00	0.01
	44	244	0.00	0.00	0.01	0.01	0.02	0.01	0.00	0.00	0.00	0.01
	45	246	0.00	0.01	0.00	0.06	0.00	0.00	0.01	0.00	0.04	0.01
	46	248	0.01	0.01	0.00	0.01	0.00	0.01	0.01	0.02	0.01	0.01
	47	250	0.02	0.01	0.00	0.02	0.01	0.00	0.00	0.00	0.00	0.01
	48	252	0.02	0.01	0.00	0.00	0.01	0.00	0.00	0.00	0.01	0.01
	49	254	0.00	0.00	0.00	0.01	0.01	0.00	0.00	0.00	0.00	0.00
	50	256	0.01	0.00	0.00	0.00	0.00	0.00	0.00	0.00	0.00	0.00
	51	258	0.00	0.00	0.00	0.01	0.00	0.00	0.00	0.00	0.00	0.00
	52	260	0.00	0.00	0.01	0.00	0.00	0.00	0.00	0.00	0.00	0.00
	53	262	0.01	0.00	0.01	0.00	0.00	0.01	0.00	0.00	0.00	0.00
	54	264	0.00	0.00	0.00	0.00	0.01	0.01	0.00	0.00	0.01	0.00
	55	268	0.00	0.00	0.00	0.00	0.00	0.00	0.00	0.01	0.00	0.00
	56	270	0.00	0.00	0.00	0.02	0.01	0.00	0.00	0.00	0.00	0.00
	57	274	0.00	0.00	0.00	0.01	0.00	0.00	0.00	0.00	0.00	0.00
	58	280	0.00	0.00	0.00	0.01	0.00	0.00	0.00	0.00	0.00	0.00
	59	292	0.01	0.00	0.00	0.00	0.00	0.00	0.00	0.00	0.00	0.00
	60	314	0.00	0.00	0.00	0.01	0.00	0.00	0.00	0.00	0.00	0.00
	61	344	0.00	0.00	0.00	0.00	0.01	0.00	0.00	0.00	0.00	0.00
SAR2.18	1	176	0.00	0.00	0.00	0.00	0.00	0.02	0.00	0.00	0.00	0.00
	2	178	0.00	0.00	0.01	0.00	0.00	0.00	0.00	0.00	0.00	0.00
	3	184	0.00	0.01	0.00	0.00	0.00	0.00	0.00	0.00	0.00	0.00
	4	186	0.00	0.00	0.00	0.00	0.00	0.01	0.00	0.00	0.00	0.00

	5	187	0.00	0.00	0.00	0.00	0.01	0.00	0.00	0.00	0.00	0.00
	6	188	0.01	0.00	0.04	0.00	0.00	0.00	0.00	0.00	0.00	0.01
	7	190	0.01	0.03	0.01	0.00	0.01	0.01	0.01	0.00	0.00	0.01
	8	192	0.02	0.01	0.02	0.04	0.00	0.02	0.01	0.07	0.00	0.02
	9	194	0.11	0.11	0.07	0.15	0.07	0.08	0.07	0.05	0.00	0.08
	10	196	0.10	0.07	0.08	0.05	0.11	0.08	0.05	0.02	0.00	0.06
	11	198	0.06	0.01	0.07	0.06	0.03	0.07	0.05	0.09	0.02	0.05
	12	200	0.05	0.07	0.09	0.01	0.04	0.04	0.03	0.02	0.01	0.04
	13	202	0.01	0.03	0.02	0.03	0.02	0.02	0.04	0.02	0.03	0.03
	14	204	0.05	0.01	0.03	0.02	0.06	0.08	0.00	0.08	0.06	0.04
	15	206	0.04	0.02	0.04	0.06	0.05	0.02	0.04	0.12	0.09	0.05
	16	208	0.01	0.04	0.02	0.05	0.03	0.03	0.05	0.07	0.10	0.05
	17	210	0.01	0.03	0.04	0.02	0.05	0.05	0.05	0.06	0.01	0.04
	18	212	0.02	0.02	0.04	0.07	0.04	0.03	0.03	0.03	0.01	0.03
	19	213	0.01	0.00	0.00	0.00	0.00	0.00	0.00	0.00	0.00	0.00
	20	214	0.05	0.01	0.03	0.05	0.04	0.00	0.02	0.06	0.08	0.04
	21	216	0.12	0.07	0.10	0.08	0.05	0.08	0.02	0.02	0.09	0.07
	22	218	0.07	0.18	0.03	0.08	0.03	0.14	0.20	0.02	0.04	0.09
	23	220	0.10	0.12	0.08	0.10	0.09	0.02	0.11	0.07	0.10	0.09
	24	222	0.10	0.07	0.07	0.01	0.05	0.03	0.03	0.00	0.03	0.05
	25	224	0.00	0.01	0.04	0.04	0.04	0.01	0.03	0.06	0.05	0.03
	26	225	0.00	0.00	0.00	0.00	0.00	0.00	0.00	0.03	0.00	0.00
	27	226	0.03	0.03	0.03	0.00	0.02	0.04	0.04	0.03	0.02	0.03
	28	228	0.00	0.01	0.00	0.00	0.00	0.01	0.00	0.00	0.02	0.00
	29	230	0.01	0.00	0.00	0.02	0.05	0.01	0.01	0.00	0.00	0.01
	30	232	0.00	0.00	0.01	0.00	0.02	0.01	0.00	0.01	0.03	0.01
	31	234	0.01	0.00	0.01	0.02	0.00	0.01	0.00	0.00	0.02	0.01
	32	236	0.00	0.00	0.01	0.02	0.01	0.00	0.00	0.00	0.01	0.01
	33	238	0.00	0.01	0.00	0.01	0.01	0.00	0.01	0.02	0.04	0.01
	34	240	0.00	0.00	0.00	0.00	0.01	0.01	0.01	0.01	0.01	0.01
	35	242	0.00	0.00	0.00	0.00	0.00	0.02	0.00	0.01	0.02	0.01
	36	244	0.00	0.00	0.01	0.01	0.01	0.02	0.01	0.00	0.00	0.01
	37	246	0.00	0.00	0.00	0.00	0.00	0.00	0.03	0.00	0.04	0.01
	38	248	0.00	0.00	0.00	0.00	0.01	0.00	0.00	0.00	0.02	0.00
	39	250	0.00	0.00	0.00	0.00	0.00	0.01	0.00	0.00	0.00	0.00
	40	258	0.00	0.00	0.00	0.00	0.00	0.00	0.01	0.00	0.00	0.00
	41	268	0.00	0.00	0.00	0.00	0.00	0.00	0.00	0.00	0.01	0.00
SAR9	1	174	0.00	0.01	0.00	0.00	0.00	0.00	0.00	0.00	0.00	0.00
	2	175	0.00	0.00	0.00	0.01	0.00	0.01	0.00	0.01	0.00	0.00
	3	176	0.00	0.00	0.00	0.03	0.00	0.02	0.00	0.00	0.00	0.01
	4	177	0.01	0.00	0.00	0.00	0.00	0.00	0.00	0.00	0.00	0.00
	5	181	0.01	0.00	0.00	0.00	0.00	0.00	0.00	0.00	0.00	0.00
	6	182	0.00	0.01	0.00	0.00	0.00	0.00	0.00	0.00	0.00	0.00
	7	183	0.01	0.01	0.00	0.00	0.00	0.02	0.00	0.00	0.00	0.00
	8	184	0.00	0.00	0.00	0.00	0.00	0.00	0.01	0.00	0.01	0.00
	9	185	0.03	0.03	0.01	0.03	0.01	0.10	0.02	0.04	0.07	0.04
	10	187	0.10	0.11	0.20	0.08	0.14	0.16	0.14	0.11	0.15	0.13
	11	189	0.12	0.07	0.10	0.08	0.14	0.07	0.11	0.08	0.08	0.09
	12	191	0.06	0.09	0.11	0.08	0.09	0.05	0.03	0.06	0.04	0.07
	13	193	0.06	0.13	0.06	0.04	0.02	0.06	0.07	0.08	0.03	0.06
	14	195	0.08	0.07	0.06	0.10	0.05	0.03	0.12	0.10	0.04	0.07
	15	198	0.03	0.04	0.06	0.09	0.16	0.08	0.10	0.08	0.09	0.08
	16	199	0.00	0.00	0.00	0.00	0.00	0.01	0.00	0.00	0.00	0.00
	17	200	0.10	0.13	0.16	0.10	0.12	0.05	0.12	0.13	0.09	0.11
	18	202	0.10	0.02	0.04	0.11	0.06	0.10	0.07	0.07	0.06	0.07

	19	203	0.00	0.01	0.00	0.00	0.00	0.00	0.00	0.00	0.00	0.00
	20	204	0.06	0.08	0.02	0.06	0.04	0.03	0.02	0.03	0.08	0.05
	21	206	0.02	0.03	0.06	0.02	0.01	0.05	0.01	0.03	0.02	0.03
	22	208	0.03	0.02	0.04	0.01	0.02	0.01	0.00	0.01	0.03	0.02
	23	210	0.06	0.05	0.01	0.03	0.02	0.00	0.03	0.02	0.03	0.03
	24	211	0.00	0.00	0.00	0.00	0.00	0.01	0.00	0.00	0.00	0.00
	25	212	0.02	0.01	0.01	0.01	0.02	0.02	0.01	0.03	0.01	0.02
	26	213	0.00	0.01	0.00	0.00	0.00	0.00	0.00	0.00	0.00	0.00
	27	214	0.02	0.01	0.00	0.01	0.00	0.01	0.03	0.02	0.02	0.01
	28	216	0.02	0.03	0.01	0.01	0.01	0.00	0.01	0.00	0.03	0.01
	29	218	0.03	0.00	0.01	0.00	0.02	0.02	0.06	0.01	0.03	0.02
	30	220	0.01	0.00	0.01	0.02	0.00	0.02	0.00	0.03	0.00	0.01
	31	222	0.00	0.01	0.02	0.01	0.00	0.02	0.01	0.01	0.01	0.01
	32	224	0.00	0.00	0.00	0.00	0.00	0.00	0.01	0.00	0.01	0.00
	33	226	0.01	0.00	0.00	0.01	0.02	0.00	0.00	0.00	0.01	0.01
	34	228	0.00	0.00	0.00	0.00	0.00	0.00	0.00	0.01	0.00	0.00
	35	232	0.00	0.01	0.00	0.00	0.00	0.00	0.00	0.00	0.00	0.00
	36	234	0.00	0.00	0.00	0.00	0.01	0.00	0.00	0.00	0.00	0.00
	37	236	0.01	0.00	0.00	0.00	0.00	0.00	0.00	0.00	0.00	0.00
	38	238	0.00	0.00	0.00	0.00	0.00	0.01	0.00	0.00	0.00	0.00
	39	241	0.00	0.00	0.00	0.00	0.00	0.00	0.01	0.00	0.00	0.00
	40	245	0.00	0.00	0.01	0.00	0.00	0.00	0.00	0.00	0.01	0.00
	41	247	0.00	0.00	0.00	0.01	0.00	0.00	0.00	0.00	0.02	0.00
	42	251	0.00	0.00	0.00	0.01	0.00	0.00	0.00	0.00	0.00	0.00
	43	253	0.00	0.00	0.00	0.00	0.02	0.00	0.00	0.00	0.00	0.00
	44	255	0.00	0.00	0.00	0.02	0.00	0.00	0.00	0.01	0.00	0.00
	45	265	0.00	0.00	0.00	0.00	0.01	0.00	0.00	0.00	0.00	0.00
	46	269	0.00	0.00	0.00	0.01	0.00	0.01	0.00	0.00	0.00	0.00
	47	273	0.00	0.00	0.00	0.00	0.00	0.01	0.00	0.01	0.00	0.00
	48	285	0.00	0.00	0.00	0.01	0.00	0.00	0.00	0.00	0.00	0.00
SAR19B3	1	118	0.01	0.00	0.00	0.00	0.00	0.00	0.00	0.00	0.00	0.00
	2	124	0.00	0.00	0.00	0.00	0.00	0.00	0.01	0.00	0.00	0.00
	3	126	0.00	0.00	0.00	0.00	0.00	0.00	0.00	0.00	0.01	0.00
	4	130	0.00	0.00	0.00	0.00	0.00	0.00	0.01	0.00	0.00	0.00
	5	132	0.01	0.01	0.00	0.00	0.00	0.00	0.00	0.00	0.00	0.00
	6	134	0.00	0.00	0.00	0.00	0.00	0.00	0.00	0.05	0.00	0.00
	7	136	0.01	0.00	0.03	0.00	0.00	0.01	0.00	0.00	0.00	0.01
	8	138	0.02	0.00	0.00	0.00	0.01	0.00	0.00	0.05	0.01	0.01
	9	140	0.00	0.01	0.00	0.05	0.00	0.00	0.03	0.00	0.02	0.01
	10	143	0.00	0.03	0.01	0.00	0.07	0.03	0.01	0.05	0.01	0.02
	11	145	0.04	0.01	0.03	0.03	0.05	0.06	0.03	0.00	0.04	0.03
	12	146	0.00	0.01	0.00	0.00	0.00	0.00	0.00	0.00	0.01	0.00
	13	147	0.02	0.07	0.05	0.02	0.01	0.01	0.06	0.06	0.02	0.04
	14	149	0.04	0.09	0.00	0.04	0.02	0.02	0.01	0.00	0.07	0.03
	15	151	0.00	0.00	0.00	0.00	0.00	0.00	0.01	0.00	0.01	0.00
	16	152	0.11	0.11	0.08	0.06	0.01	0.07	0.02	0.06	0.00	0.06
	17	153	0.04	0.07	0.10	0.04	0.05	0.07	0.05	0.05	0.02	0.06
	18	155	0.08	0.07	0.07	0.17	0.09	0.10	0.10	0.01	0.00	0.08
	19	156	0.00	0.00	0.00	0.00	0.00	0.00	0.02	0.00	0.02	0.00
	20	157	0.06	0.05	0.08	0.08	0.06	0.06	0.04	0.02	0.17	0.07
	21	159	0.07	0.04	0.05	0.11	0.14	0.06	0.10	0.11	0.03	0.08
	22	161	0.07	0.05	0.02	0.06	0.02	0.05	0.11	0.09	0.01	0.05
	23	163	0.08	0.05	0.04	0.05	0.15	0.03	0.04	0.01	0.12	0.06
	24	165	0.04	0.01	0.08	0.03	0.09	0.05	0.02	0.09	0.05	0.05
	25	167	0.02	0.02	0.02	0.00	0.03	0.03	0.06	0.03	0.03	0.03

	26	169	0.06	0.02	0.03	0.04	0.02	0.05	0.06	0.08	0.05	0.05
	27	171	0.02	0.04	0.05	0.02	0.02	0.03	0.03	0.02	0.00	0.03
	28	173	0.04	0.07	0.04	0.02	0.02	0.01	0.02	0.00	0.04	0.03
	29	174	0.00	0.00	0.00	0.00	0.01	0.00	0.00	0.01	0.00	0.00
	30	175	0.02	0.01	0.05	0.02	0.08	0.03	0.00	0.08	0.03	0.04
	31	177	0.02	0.03	0.02	0.01	0.02	0.01	0.02	0.02	0.01	0.02
	32	179	0.03	0.02	0.01	0.00	0.00	0.05	0.03	0.00	0.00	0.02
	33	181	0.00	0.00	0.01	0.02	0.00	0.04	0.01	0.00	0.01	0.01
	34	183	0.02	0.00	0.02	0.03	0.00	0.02	0.01	0.01	0.00	0.01
	35	185	0.00	0.02	0.04	0.00	0.00	0.00	0.01	0.05	0.00	0.01
	36	187	0.01	0.00	0.00	0.00	0.00	0.01	0.00	0.00	0.01	0.00
	37	189	0.01	0.01	0.00	0.01	0.00	0.04	0.00	0.02	0.00	0.01
	38	191	0.00	0.00	0.00	0.03	0.00	0.00	0.00	0.00	0.00	0.00
	39	192	0.01	0.01	0.00	0.02	0.00	0.00	0.00	0.01	0.00	0.01
	40	194	0.00	0.01	0.01	0.00	0.00	0.00	0.01	0.00	0.01	0.00
	41	196	0.00	0.01	0.00	0.00	0.00	0.00	0.00	0.00	0.03	0.00
	42	197	0.01	0.00	0.01	0.00	0.00	0.00	0.00	0.01	0.00	0.00
	43	198	0.00	0.00	0.00	0.00	0.00	0.00	0.00	0.00	0.02	0.00
	44	200	0.00	0.01	0.00	0.00	0.01	0.00	0.00	0.01	0.00	0.00
	45	201	0.00	0.00	0.00	0.00	0.00	0.00	0.00	0.00	0.01	0.00
	46	202	0.00	0.00	0.00	0.00	0.00	0.00	0.01	0.00	0.00	0.00
	47	203	0.00	0.00	0.01	0.01	0.00	0.01	0.00	0.00	0.00	0.00
	48	205	0.00	0.00	0.00	0.00	0.00	0.00	0.00	0.00	0.03	0.00
	49	206	0.00	0.00	0.00	0.00	0.01	0.00	0.00	0.00	0.00	0.00
	50	207	0.00	0.00	0.00	0.00	0.00	0.01	0.00	0.00	0.00	0.00
	51	208	0.00	0.00	0.00	0.00	0.00	0.00	0.00	0.00	0.01	0.00
	52	210	0.00	0.00	0.00	0.00	0.00	0.00	0.00	0.00	0.01	0.00
	53	212	0.00	0.01	0.00	0.00	0.00	0.00	0.00	0.00	0.00	0.00
	54	215	0.00	0.00	0.02	0.00	0.00	0.00	0.00	0.00	0.01	0.00
	55	220	0.00	0.00	0.01	0.00	0.00	0.00	0.00	0.00	0.01	0.00
	56	222	0.00	0.00	0.01	0.00	0.00	0.00	0.00	0.00	0.00	0.00
	57	223	0.00	0.00	0.00	0.00	0.00	0.00	0.00	0.00	0.01	0.00
	58	234	0.00	0.00	0.00	0.00	0.00	0.00	0.02	0.00	0.00	0.00
	59	239	0.00	0.01	0.00	0.00	0.00	0.00	0.00	0.00	0.00	0.00
	60	245	0.00	0.00	0.00	0.00	0.00	0.00	0.01	0.00	0.00	0.00
SAR19B5	1	117	0.00	0.00	0.00	0.00	0.03	0.01	0.01	0.00	0.00	0.01
	2	129	0.00	0.00	0.00	0.00	0.00	0.00	0.00	0.00	0.01	0.00
	3	133	0.00	0.00	0.00	0.00	0.00	0.00	0.00	0.02	0.00	0.00
	4	136	0.00	0.00	0.01	0.00	0.00	0.00	0.01	0.00	0.00	0.00
	5	140	0.00	0.00	0.00	0.00	0.02	0.00	0.00	0.00	0.00	0.00
	6	143	0.01	0.00	0.00	0.00	0.00	0.01	0.01	0.00	0.01	0.00
	7	145	0.00	0.00	0.03	0.00	0.00	0.00	0.01	0.00	0.00	0.00
	8	147	0.01	0.02	0.00	0.01	0.00	0.01	0.00	0.00	0.00	0.01
	9	150	0.02	0.00	0.01	0.01	0.00	0.00	0.00	0.00	0.01	0.01
	10	152	0.04	0.05	0.00	0.03	0.01	0.04	0.00	0.01	0.02	0.02
	11	154	0.05	0.02	0.00	0.04	0.00	0.01	0.01	0.05	0.00	0.02
	12	156	0.01	0.01	0.01	0.00	0.00	0.00	0.03	0.00	0.01	0.01
	13	158	0.02	0.03	0.00	0.00	0.00	0.03	0.02	0.02	0.03	0.02
	14	159	0.00	0.00	0.01	0.00	0.00	0.00	0.00	0.00	0.00	0.00
	15	160	0.00	0.04	0.01	0.02	0.05	0.02	0.01	0.00	0.01	0.02
	16	163	0.01	0.03	0.05	0.00	0.05	0.02	0.00	0.05	0.00	0.02
	17	165	0.10	0.02	0.02	0.10	0.03	0.03	0.05	0.05	0.02	0.05
	18	167	0.02	0.05	0.02	0.03	0.02	0.04	0.01	0.01	0.04	0.03
	19	169	0.03	0.04	0.04	0.02	0.02	0.03	0.03	0.04	0.04	0.03
	20	170	0.00	0.00	0.00	0.00	0.00	0.00	0.01	0.00	0.00	0.00

21	171	0.03	0.00	0.04	0.05	0.03	0.00	0.00	0.04	0.03	0.03
22	173	0.02	0.03	0.05	0.00	0.04	0.01	0.00	0.02	0.02	0.02
23	175	0.05	0.05	0.01	0.06	0.04	0.00	0.01	0.04	0.00	0.03
24	177	0.00	0.00	0.03	0.00	0.05	0.02	0.02	0.05	0.02	0.02
25	179	0.03	0.01	0.03	0.03	0.00	0.03	0.03	0.02	0.01	0.02
26	182	0.02	0.00	0.02	0.02	0.01	0.03	0.05	0.02	0.00	0.02
27	183	0.03	0.03	0.03	0.03	0.05	0.05	0.00	0.05	0.04	0.04
28	186	0.04	0.04	0.00	0.04	0.01	0.03	0.04	0.01	0.02	0.03
29	188	0.03	0.03	0.04	0.00	0.01	0.01	0.04	0.01	0.03	0.02
30	190	0.01	0.02	0.03	0.00	0.01	0.00	0.04	0.02	0.03	0.02
31	192	0.02	0.02	0.02	0.00	0.00	0.02	0.02	0.00	0.01	0.01
32	194	0.01	0.02	0.04	0.02	0.07	0.01	0.02	0.02	0.02	0.03
33	196	0.01	0.08	0.05	0.02	0.04	0.00	0.05	0.02	0.04	0.04
34	198	0.03	0.01	0.01	0.02	0.00	0.04	0.02	0.04	0.01	0.02
35	200	0.01	0.00	0.01	0.01	0.06	0.02	0.02	0.00	0.02	0.02
36	202	0.04	0.02	0.01	0.01	0.00	0.05	0.01	0.02	0.00	0.02
37	204	0.01	0.03	0.00	0.03	0.03	0.02	0.04	0.05	0.02	0.03
38	206	0.01	0.01	0.02	0.03	0.01	0.00	0.02	0.04	0.00	0.02
39	208	0.02	0.01	0.04	0.03	0.02	0.00	0.01	0.01	0.01	0.02
40	210	0.01	0.00	0.06	0.02	0.03	0.00	0.01	0.01	0.04	0.02
41	212	0.01	0.02	0.03	0.02	0.00	0.03	0.01	0.00	0.01	0.02
42	214	0.02	0.01	0.01	0.01	0.01	0.05	0.03	0.01	0.02	0.02
43	216	0.00	0.01	0.01	0.02	0.00	0.04	0.01	0.01	0.02	0.01
44	218	0.01	0.01	0.01	0.04	0.04	0.00	0.03	0.01	0.00	0.02
45	220	0.00	0.02	0.01	0.02	0.05	0.02	0.03	0.02	0.04	0.03
46	223	0.00	0.00	0.00	0.03	0.01	0.02	0.01	0.02	0.02	0.01
47	225	0.02	0.01	0.02	0.00	0.01	0.02	0.01	0.00	0.01	0.01
48	226	0.00	0.01	0.01	0.02	0.00	0.03	0.00	0.02	0.03	0.01
49	229	0.01	0.01	0.00	0.00	0.02	0.01	0.00	0.00	0.00	0.01
50	231	0.03	0.00	0.01	0.01	0.01	0.02	0.00	0.00	0.00	0.01
51	233	0.01	0.01	0.01	0.03	0.00	0.00	0.00	0.00	0.03	0.01
52	235	0.01	0.00	0.02	0.00	0.00	0.00	0.00	0.00	0.04	0.01
53	237	0.00	0.03	0.01	0.01	0.00	0.01	0.01	0.00	0.01	0.01
54	239	0.02	0.00	0.00	0.00	0.01	0.00	0.00	0.02	0.04	0.01
55	241	0.01	0.01	0.01	0.02	0.00	0.00	0.02	0.01	0.00	0.01
56	243	0.00	0.00	0.00	0.00	0.00	0.03	0.00	0.01	0.00	0.00
57	245	0.01	0.01	0.02	0.00	0.00	0.02	0.01	0.00	0.01	0.01
58	247	0.01	0.00	0.00	0.01	0.01	0.00	0.00	0.00	0.00	0.00
59	249	0.01	0.00	0.02	0.01	0.00	0.00	0.01	0.01	0.00	0.01
60	251	0.01	0.00	0.00	0.00	0.00	0.02	0.02	0.00	0.02	0.01
61	253	0.01	0.00	0.00	0.00	0.01	0.01	0.00	0.01	0.00	0.00
62	254	0.00	0.00	0.00	0.00	0.00	0.00	0.00	0.01	0.00	0.00
63	256	0.00	0.01	0.01	0.00	0.01	0.00	0.00	0.01	0.02	0.01
64	257	0.00	0.01	0.00	0.00	0.00	0.00	0.01	0.00	0.01	0.00
65	260	0.00	0.00	0.00	0.01	0.00	0.00	0.00	0.00	0.01	0.00
66	262	0.00	0.01	0.00	0.00	0.00	0.00	0.00	0.00	0.00	0.00
67	264	0.00	0.00	0.00	0.00	0.00	0.00	0.00	0.01	0.00	0.00
68	266	0.00	0.01	0.00	0.00	0.00	0.00	0.00	0.00	0.01	0.00
69	268	0.00	0.00	0.00	0.00	0.00	0.00	0.00	0.00	0.01	0.00
70	270	0.00	0.01	0.00	0.00	0.00	0.00	0.00	0.00	0.00	0.00
71	272	0.00	0.00	0.00	0.00	0.01	0.00	0.00	0.01	0.00	0.00
72	276	0.00	0.00	0.00	0.01	0.00	0.00	0.00	0.00	0.01	0.00
73	282	0.00	0.00	0.00	0.00	0.00	0.00	0.01	0.00	0.00	0.00
74	284	0.00	0.00	0.00	0.00	0.00	0.00	0.02	0.00	0.00	0.00
75	287	0.00	0.00	0.00	0.00	0.00	0.00	0.01	0.02	0.00	0.00

	76	288	0.00	0.00	0.01	0.00	0.00	0.00	0.00	0.00	0.00	0.00
	77	303	0.00	0.00	0.01	0.00	0.00	0.00	0.00	0.00	0.00	0.00
	78	320	0.00	0.00	0.00	0.00	0.00	0.00	0.01	0.00	0.00	0.00
	79	331	0.00	0.00	0.00	0.00	0.00	0.01	0.00	0.00	0.00	0.00
	80	337	0.00	0.00	0.00	0.00	0.00	0.01	0.00	0.00	0.00	0.00
	81	349	0.00	0.00	0.00	0.01	0.00	0.00	0.00	0.00	0.00	0.00
SARA2F	1	179	0.00	0.00	0.00	0.00	0.00	0.00	0.01	0.00	0.00	0.00
	2	181	0.00	0.00	0.00	0.00	0.00	0.01	0.00	0.00	0.00	0.00
	3	183	0.00	0.00	0.00	0.01	0.02	0.02	0.00	0.00	0.00	0.01
	4	185	0.01	0.00	0.00	0.00	0.00	0.00	0.00	0.00	0.00	0.00
	5	187	0.00	0.00	0.01	0.00	0.00	0.00	0.00	0.01	0.01	0.00
	6	189	0.00	0.00	0.01	0.00	0.00	0.03	0.00	0.00	0.04	0.01
	7	191	0.01	0.03	0.07	0.07	0.09	0.11	0.10	0.15	0.14	0.09
	8	193	0.03	0.00	0.01	0.00	0.02	0.05	0.10	0.05	0.04	0.03
	9	195	0.00	0.02	0.01	0.02	0.03	0.04	0.03	0.15	0.04	0.04
	10	197	0.02	0.01	0.05	0.00	0.01	0.03	0.03	0.06	0.05	0.03
	11	199	0.01	0.04	0.04	0.03	0.01	0.03	0.02	0.03	0.03	0.03
	12	201	0.03	0.03	0.08	0.02	0.03	0.04	0.06	0.01	0.01	0.04
	13	203	0.00	0.00	0.01	0.00	0.00	0.00	0.00	0.00	0.00	0.00
	14	204	0.01	0.03	0.04	0.05	0.05	0.01	0.02	0.03	0.04	0.03
	15	205	0.18	0.10	0.12	0.09	0.11	0.06	0.10	0.06	0.04	0.10
	16	207	0.08	0.09	0.04	0.05	0.09	0.04	0.04	0.08	0.09	0.07
	17	209	0.09	0.08	0.09	0.10	0.10	0.08	0.10	0.09	0.14	0.10
	18	211	0.03	0.03	0.07	0.09	0.07	0.01	0.04	0.05	0.00	0.04
	19	212	0.00	0.00	0.00	0.04	0.00	0.00	0.00	0.00	0.00	0.00
	20	213	0.01	0.07	0.10	0.07	0.08	0.08	0.04	0.03	0.03	0.06
	21	215	0.03	0.03	0.00	0.07	0.04	0.01	0.06	0.02	0.05	0.04
	22	218	0.03	0.04	0.01	0.03	0.03	0.09	0.02	0.02	0.06	0.04
	23	220	0.03	0.00	0.02	0.04	0.03	0.00	0.01	0.02	0.00	0.02
	24	222	0.02	0.02	0.00	0.01	0.00	0.01	0.03	0.01	0.01	0.01
	25	224	0.01	0.02	0.02	0.00	0.02	0.01	0.01	0.00	0.03	0.01
	26	225	0.00	0.00	0.00	0.00	0.00	0.00	0.00	0.00	0.01	0.00
	27	226	0.01	0.00	0.03	0.03	0.00	0.03	0.02	0.01	0.03	0.02
	28	227	0.00	0.00	0.00	0.00	0.00	0.01	0.00	0.00	0.00	0.00
	29	228	0.03	0.04	0.03	0.00	0.03	0.01	0.02	0.03	0.01	0.02
	30	230	0.02	0.04	0.00	0.00	0.03	0.03	0.01	0.02	0.03	0.02
	31	232	0.00	0.01	0.00	0.01	0.00	0.00	0.01	0.00	0.01	0.00
	32	234	0.00	0.02	0.00	0.04	0.03	0.01	0.00	0.01	0.02	0.02
	33	236	0.01	0.03	0.03	0.03	0.02	0.00	0.01	0.00	0.00	0.02
	34	238	0.01	0.02	0.00	0.01	0.00	0.00	0.01	0.01	0.01	0.01
	35	240	0.03	0.01	0.00	0.00	0.00	0.02	0.01	0.00	0.00	0.01
	36	242	0.02	0.01	0.02	0.00	0.01	0.00	0.00	0.00	0.00	0.01
	37	244	0.02	0.02	0.01	0.01	0.00	0.01	0.00	0.01	0.00	0.01
	38	246	0.07	0.01	0.02	0.02	0.01	0.00	0.00	0.00	0.00	0.01
	39	248	0.04	0.01	0.02	0.01	0.01	0.00	0.01	0.00	0.00	0.01
	40	250	0.01	0.01	0.01	0.00	0.00	0.00	0.00	0.00	0.00	0.00
	41	253	0.00	0.00	0.01	0.00	0.00	0.00	0.01	0.00	0.00	0.00
	42	255	0.02	0.01	0.01	0.00	0.00	0.01	0.00	0.00	0.00	0.01
	43	257	0.00	0.01	0.00	0.00	0.00	0.03	0.00	0.00	0.00	0.00
	44	259	0.00	0.02	0.00	0.00	0.00	0.02	0.02	0.00	0.00	0.01
	45	261	0.01	0.00	0.00	0.00	0.00	0.00	0.00	0.00	0.00	0.00
	46	263	0.00	0.00	0.01	0.00	0.00	0.01	0.01	0.00	0.00	0.00
	47	265	0.00	0.02	0.00	0.01	0.00	0.01	0.00	0.00	0.00	0.00
	48	267	0.01	0.00	0.00	0.00	0.00	0.00	0.00	0.00	0.00	0.00
	49	269	0.01	0.00	0.00	0.00	0.00	0.00	0.00	0.00	0.00	0.00

	50	271	0.01	0.00	0.00	0.00	0.00	0.00	0.00	0.00	0.00	0.00
	51	273	0.00	0.01	0.00	0.01	0.00	0.00	0.00	0.00	0.00	0.00
	52	286	0.00	0.00	0.00	0.00	0.01	0.00	0.00	0.00	0.00	0.00
	53	297	0.00	0.01	0.00	0.00	0.00	0.00	0.00	0.00	0.00	0.00
	54	302	0.00	0.01	0.00	0.00	0.00	0.00	0.00	0.00	0.00	0.00
SARA3C	1	101	0.01	0.00	0.00	0.00	0.00	0.00	0.00	0.00	0.00	0.00
	2	105	0.00	0.00	0.00	0.00	0.00	0.00	0.00	0.02	0.00	0.00
	3	110	0.01	0.00	0.00	0.00	0.00	0.00	0.00	0.00	0.00	0.00
	4	116	0.00	0.01	0.00	0.00	0.01	0.01	0.00	0.00	0.00	0.00
	5	120	0.00	0.00	0.00	0.00	0.00	0.00	0.00	0.00	0.02	0.00
	6	122	0.02	0.00	0.02	0.00	0.00	0.00	0.00	0.02	0.01	0.01
	7	123	0.00	0.01	0.00	0.00	0.00	0.00	0.02	0.00	0.00	0.00
	8	124	0.02	0.02	0.01	0.00	0.00	0.00	0.00	0.00	0.00	0.01
	9	125	0.00	0.02	0.00	0.00	0.00	0.03	0.02	0.00	0.03	0.01
	10	126	0.00	0.01	0.00	0.00	0.00	0.00	0.02	0.01	0.00	0.00
	11	128	0.00	0.01	0.00	0.00	0.01	0.00	0.00	0.01	0.00	0.00
	12	129	0.10	0.06	0.08	0.02	0.12	0.02	0.02	0.16	0.03	0.07
	13	130	0.00	0.00	0.04	0.00	0.00	0.00	0.00	0.01	0.00	0.01
	14	132	0.05	0.06	0.03	0.03	0.05	0.11	0.07	0.07	0.09	0.06
	15	133	0.04	0.05	0.01	0.06	0.03	0.06	0.08	0.09	0.13	0.06
	16	134	0.00	0.00	0.00	0.00	0.00	0.01	0.00	0.00	0.00	0.00
	17	136	0.02	0.02	0.01	0.03	0.07	0.02	0.03	0.00	0.03	0.03
	18	137	0.00	0.00	0.00	0.00	0.00	0.01	0.01	0.00	0.00	0.00
	19	138	0.02	0.00	0.03	0.09	0.03	0.03	0.00	0.02	0.07	0.03
	20	139	0.00	0.02	0.00	0.02	0.00	0.00	0.00	0.00	0.00	0.00
	21	140	0.02	0.01	0.02	0.02	0.03	0.04	0.00	0.02	0.02	0.02
	22	143	0.01	0.00	0.01	0.01	0.01	0.02	0.01	0.01	0.01	0.01
	23	144	0.00	0.00	0.00	0.00	0.00	0.00	0.03	0.00	0.00	0.00
	24	145	0.00	0.00	0.02	0.02	0.00	0.00	0.03	0.02	0.03	0.01
	25	146	0.00	0.00	0.00	0.00	0.00	0.00	0.00	0.00	0.02	0.00
	26	147	0.04	0.02	0.01	0.02	0.02	0.04	0.01	0.03	0.00	0.02
	27	148	0.00	0.00	0.00	0.00	0.00	0.00	0.01	0.00	0.00	0.00
	28	150	0.00	0.02	0.02	0.02	0.01	0.05	0.02	0.02	0.03	0.02
	29	152	0.00	0.02	0.02	0.02	0.01	0.03	0.02	0.00	0.00	0.01
	30	153	0.00	0.02	0.00	0.00	0.00	0.00	0.00	0.00	0.00	0.00
	31	154	0.00	0.00	0.00	0.00	0.00	0.01	0.00	0.00	0.00	0.00
	32	155	0.02	0.01	0.00	0.02	0.03	0.01	0.01	0.01	0.00	0.01
	33	157	0.02	0.01	0.03	0.00	0.03	0.00	0.02	0.01	0.01	0.02
	34	159	0.00	0.00	0.00	0.02	0.01	0.00	0.00	0.00	0.03	0.01
	35	161	0.02	0.03	0.07	0.00	0.03	0.02	0.01	0.00	0.00	0.02
	36	162	0.00	0.00	0.02	0.00	0.00	0.00	0.00	0.00	0.00	0.00
	37	163	0.00	0.00	0.01	0.01	0.01	0.04	0.00	0.01	0.00	0.01
	38	165	0.02	0.00	0.01	0.03	0.04	0.03	0.01	0.02	0.01	0.02
	39	166	0.00	0.00	0.01	0.00	0.00	0.01	0.00	0.00	0.01	0.00
	40	167	0.04	0.00	0.10	0.01	0.02	0.05	0.02	0.00	0.09	0.04
	41	168	0.00	0.00	0.00	0.00	0.00	0.02	0.00	0.00	0.00	0.00
	42	169	0.00	0.01	0.02	0.06	0.00	0.03	0.01	0.00	0.01	0.02
	43	172	0.02	0.05	0.00	0.05	0.04	0.00	0.03	0.01	0.00	0.02
	44	173	0.00	0.00	0.00	0.02	0.00	0.00	0.00	0.00	0.00	0.00
	45	174	0.04	0.00	0.03	0.01	0.02	0.03	0.03	0.04	0.00	0.02
	46	175	0.02	0.00	0.00	0.00	0.00	0.00	0.00	0.00	0.00	0.00
	47	176	0.00	0.00	0.01	0.06	0.02	0.00	0.05	0.00	0.02	0.02
	48	178	0.01	0.01	0.05	0.04	0.03	0.00	0.04	0.08	0.02	0.03
	49	179	0.00	0.00	0.00	0.00	0.00	0.00	0.00	0.00	0.01	0.00
	50	180	0.03	0.01	0.06	0.03	0.04	0.01	0.00	0.01	0.01	0.02

51	181	0.00	0.01	0.00	0.00	0.00	0.01	0.00	0.00	0.00	0.00
52	182	0.01	0.03	0.01	0.00	0.03	0.03	0.04	0.07	0.04	0.03
53	183	0.01	0.00	0.00	0.01	0.00	0.00	0.00	0.00	0.00	0.00
54	184	0.02	0.06	0.03	0.02	0.03	0.02	0.04	0.01	0.03	0.03
55	185	0.01	0.00	0.00	0.01	0.00	0.00	0.00	0.01	0.01	0.00
56	186	0.05	0.03	0.02	0.03	0.02	0.00	0.02	0.00	0.02	0.02
57	187	0.00	0.00	0.00	0.00	0.02	0.00	0.00	0.00	0.00	0.00
58	188	0.03	0.00	0.01	0.04	0.00	0.01	0.00	0.01	0.04	0.02
59	190	0.04	0.02	0.02	0.00	0.00	0.02	0.02	0.00	0.02	0.02
60	191	0.00	0.00	0.00	0.00	0.00	0.02	0.00	0.00	0.00	0.00
61	192	0.00	0.02	0.00	0.00	0.02	0.00	0.04	0.03	0.01	0.01
62	193	0.00	0.02	0.01	0.00	0.00	0.00	0.02	0.00	0.00	0.01
63	194	0.00	0.00	0.02	0.02	0.00	0.03	0.01	0.01	0.00	0.01
64	195	0.00	0.00	0.00	0.00	0.02	0.00	0.00	0.00	0.00	0.00
65	196	0.00	0.00	0.02	0.00	0.00	0.00	0.01	0.06	0.00	0.01
66	197	0.00	0.00	0.01	0.00	0.00	0.00	0.00	0.00	0.01	0.00
67	198	0.02	0.00	0.02	0.01	0.00	0.01	0.00	0.00	0.00	0.01
68	200	0.01	0.02	0.00	0.00	0.00	0.00	0.02	0.02	0.00	0.01
69	202	0.00	0.00	0.01	0.00	0.01	0.00	0.00	0.00	0.00	0.00
70	203	0.00	0.00	0.00	0.00	0.00	0.01	0.00	0.00	0.00	0.00
71	204	0.00	0.00	0.00	0.00	0.00	0.00	0.00	0.02	0.00	0.00
72	205	0.00	0.02	0.01	0.01	0.00	0.00	0.00	0.00	0.00	0.00
73	206	0.01	0.00	0.01	0.01	0.01	0.00	0.01	0.01	0.00	0.01
74	208	0.00	0.01	0.00	0.00	0.00	0.00	0.00	0.00	0.00	0.00
75	209	0.00	0.00	0.02	0.00	0.00	0.00	0.02	0.00	0.00	0.00
76	213	0.02	0.01	0.00	0.00	0.00	0.00	0.01	0.00	0.00	0.00
77	215	0.00	0.00	0.00	0.01	0.00	0.02	0.00	0.00	0.00	0.00
78	217	0.00	0.01	0.00	0.01	0.00	0.01	0.00	0.00	0.00	0.00
79	220	0.00	0.00	0.00	0.00	0.00	0.02	0.00	0.00	0.00	0.00
80	221	0.01	0.03	0.01	0.00	0.00	0.00	0.00	0.00	0.02	0.01
81	223	0.00	0.01	0.00	0.00	0.02	0.00	0.00	0.02	0.00	0.01
82	225	0.00	0.02	0.00	0.02	0.01	0.01	0.02	0.00	0.02	0.01
83	229	0.00	0.00	0.00	0.02	0.00	0.00	0.00	0.00	0.00	0.00
84	231	0.01	0.00	0.01	0.01	0.01	0.00	0.02	0.00	0.00	0.01
85	233	0.00	0.02	0.00	0.00	0.00	0.00	0.01	0.00	0.00	0.00
86	235	0.01	0.01	0.00	0.01	0.02	0.00	0.00	0.00	0.00	0.01
87	237	0.01	0.01	0.00	0.00	0.00	0.00	0.00	0.00	0.00	0.00
88	242	0.00	0.00	0.00	0.00	0.00	0.00	0.00	0.00	0.01	0.00
89	244	0.00	0.01	0.01	0.00	0.00	0.00	0.00	0.00	0.00	0.00
90	248	0.00	0.02	0.00	0.00	0.00	0.00	0.00	0.00	0.00	0.00
91	266	0.01	0.00	0.00	0.00	0.00	0.00	0.00	0.00	0.00	0.00
92	275	0.00	0.01	0.00	0.00	0.00	0.00	0.00	0.00	0.00	0.00
93	293	0.04	0.00	0.00	0.00	0.00	0.00	0.00	0.00	0.00	0.00
94	311	0.01	0.00	0.00	0.00	0.00	0.00	0.00	0.00	0.00	0.00

* N_A= number of alleles per locus.
